# Erdosteine Enhances Tobramycin Activity Against a Tobramycin-Resistant Clinical Isolate and PAO1 Reference Strain of *Pseudomonas aeruginosa*

**DOI:** 10.1007/s00408-026-00951-6

**Published:** 2026-11-09

**Authors:** Bailey Ramsden, Cristina Cigana, Davide Gugliandolo, Martina Rossano, Alessandra Bragonzi, Clive P. Page, Richard T. Amison

**Affiliations:** 1 Unit of Pulmonary Pharmacology, Institute of Pharmaceutical Science, School of Cancer & Pharmaceutical Sciences, King’s College London, 5.72 Franklin-Wilkins Building, Waterloo Campus, London, SE1 9NH UK; 2 Infections and Cystic Fibrosis Unit, Division of Immunology, Transplantation and Infectious Diseases, IRCCS San Raffaele Institute, Milan, Italy

**Keywords:** *Pseudomonas*, *Pseudomonas aeruginosa*, Lung infections, Erdosteine, Tobramycin, Antibiotic resistance

## Abstract

**Background:**

Chronic respiratory infections caused by pathogens including *Pseudomonas aeruginosa* (*P. aeruginosa*) pose a major therapeutic challenge in patients with pulmonary diseases such as cystic fibrosis (CF) and non-CF bronchiectasis, where treatment efficacy is limited by the formation of biofilms, the emergence of multidrug resistant pathogens and a shortage of new and effective antibiotic classes. One approach to address this problem involves the use of “antibiotic enhancers”, which are compounds that potentiate the activity of existing antibiotics without possessing direct antimicrobial effects of their own. The mucoactive thiol-based prodrug erdosteine, currently approved for the treatment of acute and chronic respiratory diseases, has been reported to exhibit anti-adhesive activity against respiratory pathogens, suggesting a clinically relevant contribution to the prevention of bacterial colonisation. Furthermore, in addition to reported anti-inflammatory and antioxidant activities, erdosteine has demonstrated the ability to potentiate the activity of various antimicrobial drugs. However, to date, whether erdosteine can enhance the activity of tobramycin, an antibiotic widely used to treat pathogens in the respiratory tract, has not been investigated. In this study, we investigated the ability of MET-1, the primary active metabolite of erdosteine, to enhance the activity of tobramycin against *P. aeruginosa* strains using a panel of both in vitro and in vivo assays.

**Methods:**

The activity of both mono- and combination therapies was measured in planktonic *P. aeruginosa* (strains RP73 & PAO1) using in vitro microdilution assays including chequerboard analysis and time-kill assays, whilst activity against biofilms were analysed using microtiter plate assays with readouts including total biomass, metabolic activity, and bacterial enumeration. Combination therapy of 40 mg/kg tobramycin and 100 mg/kg erdosteine was also assessed in vivo using a murine model of chronic pulmonary infection induced by *P. aeruginosa.*

**Results:**

10 mg/L MET-1 significantly enhanced the antimicrobial activity of tobramycin in planktonic cultures of RP73 at 24 h and PAO1 at 4 h, leading to a greater than 2 log_10_ reduction in bacterial numbers compared to tobramycin monotherapy, despite MET-1 demonstrating no intrinsic bactericidal activity. In mature biofilms, MET-1 substantially enhanced tobramycin activity, reducing the biomass-derived IC₅₀ by approximately 18-fold in RP73 and 13-fold in PAO1. Finally, combination therapy of 40 mg/kg tobramycin and 100 mg/kg erdosteine was associated with a lower pulmonary bacterial burden in a murine model of chronic pulmonary infection.

**Conclusions:**

Our in vitro and in vivo studies highlight the ability of erdosteine to potentiate the activity of tobramycin suggesting that erdosteine has the potential to reduce the burden of long-term antimicrobial therapies in patients with chronic respiratory infections and thus improve clinical outcomes.

**Supplementary Information:**

The online version contains supplementary material available at https://doi.org/10.1007/s00408-026-00951-6.

## Introduction

Chronic respiratory diseases such as chronic obstructive pulmonary disease (COPD), cystic fibrosis (CF), and chronic bronchitis (CB) are often associated with persistent inflammation leading to chronic lung damage, impaired mucociliary clearance and dysregulated immune responses preventing effective pathogen clearance. As a result, these diseases are associated with chronic bacterial colonisation of the small airways by species of the genera *Staphylococcus* and *Pseudomonas* [1]. Furthermore, these patients are also associated with frequent acute infective pulmonary exacerbations (PEx), which are known to be a primary cause behind accelerated lung function decline and disease severity [2–4]. Treatment of these chronic infections is limited by the formation of bacterial biofilms, where the formation of extracellular matrix structures associated with mature biofilms shields pathogens from both host immune responses, and limits the penetration and activity of conventional antimicrobial therapies [5, 6]. Treatment is further complicated by the rise of multidrug-resistant (MDR) and extensively drug-resistant (XDR) pathogens [7–10].

Tobramycin remains a primary therapy for *Pseudomonas aeruginosa* (*P. aeruginosa*) infections, administered as an inhaled formulation for the treatment of chronic infections [11] or intravenously for acute exacerbations. However, the growing prevalence of tobramycin-resistant *P. aeruginosa* strains, coupled with the limited availability of novel antibiotic classes, has made these infections increasingly challenging to manage. One approach to overcome bacterial resistance has focused on adjunctive therapies that can enhance or restore antimicrobial activity of existing antibiotics.

The orally active thiol-derived pro-drug erdosteine is a licenced medicine used internationally to treat acute and chronic respiratory diseases, including acute episodes of bronchitis. Whilst originally licenced as a mucolytic, there is both pre-clinical and clinical evidence for a multimodal mechanism of action for erdosteine including mucoactive, antioxidant, anti-inflammatory, antiviral and antibiotic enhancing activities [12–14]. Significant therapeutic benefits of erdosteine have been reported, with its use as an adjunct therapy reducing mucus production, sputum viscosity, and reactive oxygen species (ROS), leading to reduced dyspnoea and increased FEV1 in patients with COPD and CB [15–17]. Significantly, multiple clinical studies have now demonstrated that chronic erdosteine therapy reduces both duration and frequency of PEx [18–21].

One previous concern for the use of thiol-derived mucolytic agents, particularly carbocysteine and N-acetylcysteine (NAC), is their potential to interact with multiple classes of antibiotics, leading to reduced antimicrobial activity through drug-drug interactions [22]. However, structural differences between erdosteine and the other thiol-derivatives appear to remove this negative interaction. Indeed, rather than compromising activity, erdosteine has demonstrated numerous antibiotic-enhancing capabilities. In vitro studies using the active metabolite N-thiodiglycolyl-homocysteine (MET-1) have demonstrated reduced bacterial adhesion to lung epithelial cells and improved antimicrobial activity of vancomycin, linezolid, amoxicillin and levofloxacin in *Staphylococcus aureus* (*S. aureus*) biofilms through enhanced drug penetration. In fact, erdosteine has been shown to possess anti-adhesive properties, as demonstrated by Braga et al. with evidence of reduced bacterial adherence to epithelial cells, supporting a potential role in limiting colonization and infection [23–25]. Importantly, this has also been reported clinically through enhanced clinical activity of amoxicillin in CB patients. A meta-analysis of 7 randomised clinical trials (RCTs) revealed that erdosteine plus antibiotics was more effective than antibiotic monotherapy in patients with CB/COPD for the treatment of acute infective PEx [26, 27]. However, despite the ability of erdosteine to improve the activity of different classes of antibiotics against gram-positive pathogens such as *S. aureus* [25], its ability to potentiate standard-of-care (SoC) antibiotic therapy against gram-negative pathogens such as *P. aeruginosa*, which typically cause respiratory infections, has not been explored. We therefore set out to investigate the potential benefits of erdosteine in combination with tobramycin against two *P. aeruginosa* isolates: (1) RP73, a MDR clinical isolate associated with chronic infections [28], and (2) PAO1, a well-characterised laboratory reference strain commonly used to study pathogenesis and biofilm formation [29]. We combined conventional in vitro assays of bactericidal activity such as the chequerboard assay of synergy, bacterial time-kill and biofilm microtiter assays coupled with an in vivo murine model of chronic pulmonary infection with *P. aeruginosa*.

## Materials and Methods

### In Vitro Bacterial Culture Conditions and Maintenance

All bacterial strains were stored at -80 °C using the CryoBead system and routinely maintained using Tryptone Soy Agar (TSA) made using 15 g/L Difco™ Agar Bacteriological (Thermo Fisher Scientific Ltd., UK; 113–1496) and 30 g/L Tryptic Soy Broth (Thermo Fisher Scientific Ltd., UK; CM0129B). Two *P. aeruginosa* strains were provided by Dr Alessandra Bragonzi at the Infection and Cystic Fibrosis Unit, San Raffaele Scientific Institute, Milan, Italy. The fully sequenced tobramycin-sensitive laboratory reference strain, PAO1, and a multi-drug resistant non-mucoid strain, RP73, isolated 17.5 years post onset of infection in a CF patient [28, 29]. MDR was defined as acquired non-susceptibility to at least one antimicrobial agent in three or more antimicrobial classes. For chequerboard and time-kill assays, bacteria were grown in tryptic soy broth (TSB), whilst in biofilm studies TSB was supplemented with 0.25% glucose (D-glucose anhydrous, Thermo Fisher Scientific Ltd., UK; G/0500/53) and 0.5% NaCl (Thermo Fisher Scientific Ltd., UK; A12313.0G) to promote biofilm formation as previously reported [25]. For in vivo studies, RP73 was grown on TSA and cultured in nutrient broth at 37 °C, 120 rpm.

### Drug Preparation

Erdosteine and its primary active metabolite, MET-1, were kindly donated by Edmond Pharma (Milan, Italy). MET-1 (97.5% purity by HPLC) and erdosteine (99.3% purity by HPLC) were dissolved in 100% DMSO at 10 g/L and filter sterilised through a 0.20 μm membrane (Cambridge Bioscience Ltd., Cambridge, UK). Matched DMSO controls were included at equivalent solvent concentrations. Tobramycin (Sigma-Aldrich, Dorset, UK; PHR1079) was dissolved in PBS at 10 g/L and filter sterilised through a 0.20 μm membrane (Cambridge Bioscience Ltd., Cambridge, UK). For in vivo studies, erdosteine was dissolved in DMEM and adjusted to pH 7.4. Tobramycin (Fisher Scientific) stocks were prepared in PBS at 1 g/L and filter sterilised (0.22 μm). All stock solutions were stored at -20 °C and were single-use once thawed.

### Minimum Inhibitory Concentration (MIC) Assay

MET-1, erdosteine and tobramycin MICs were calculated for RP73 and PAO1. MICs were determined at the growth/no-growth interface following EUCAST methodology as previously reported [30]. Briefly, serial 2-fold dilutions of the drug (100µL) were prepared in TSB in flat-well polycarbonate 96 well plates and treated with (100µL) 1 × 10^6^ cfu/mL, giving a final inoculum of 5 × 10⁵ cfu/mL in a final volume of 200µL. Drug-free growth controls and uninoculated sterility controls were included on each plate. The highest MET-1 concentrations tested, 1024 and 2048 mg/L, corresponded to final DMSO concentrations of 10.24% and 20.48% (v/v), respectively. Plates were sealed with a breathable membrane and incubated in a humidified chamber at 37 °C for 16–20 h. Resazurin sodium salt was added to a final concentration of 0.15 mM and plates were incubated for an additional 2–4 h. Resazurin is a blue-coloured dye that is reduced by aerobic respiration of metabolically active cells to the pink-coloured fluorescent resorufin [30, 31]. All MICs were read against matched growth and no-growth controls to standardise visual interpretation between plates. Wells showing an intermediate purple-to-pink colour change were interpreted as demonstrating residual metabolic activity and were therefore not classified as inhibited.

### Chequerboard Assay of Synergy

As erdosteine is a prodrug, for all in vitro efficacy assays, we used the primary active metabolite MET-1. MET-1-tobramycin combinations were prepared in TSB in 96-well plates. The starting concentration for tobramycin was 2x MIC and, given that no MIC could be determined for MET-1, the highest soluble concentration of MET-1 was used (1024 mg/L). A double serial 2-fold dilution of each drug in PBS was then performed in sterile flat well polycarbonate plates creating a total of 64 different combinations per pair. Plates were sealed with a breathable membrane and incubated in a humidified chamber at 37 °C for 16–20 h. Resazurin sodium salt was added to a final concentration of 0.15 mM and plates were incubated for an additional 2–4 h. Given the lack of MIC for MET-1, the Fractional Inhibitory Concentration Indices (FICI) could not be calculated. Instead, the effect of the combination was determined based on the difference between the MIC of tobramycin as a monotherapy and in combination with MET-1 using a growth/no-growth interface.

### Bacterial Time-Kill Assays

RP73 or PAO1 at 5 × 10^5^ cfu/mL were cultured in the presence of vehicle, tobramycin, MET-1 (10 mg/L), or tobramycin in combination with either 2, 5 or 10 mg/L MET-1. MET-1 was used at concentrations in the same order of magnitude of the peak blood concentrations obtainable at the approved therapeutic doses [23, 24]. MET-1 concentrations corresponded to final DMSO concentrations of 0.02%, 0.05% and 0.10% (v/v), with matched DMSO-only controls included. Tobramycin was used at concentrations 0.5, 1 and 4 times the MIC as determined by the methodology described above. Bacterial viability was determined by aliquot collection at 0, 2-, 4-, 7- and 24-hours post-inoculation. Samples of bacterial suspensions were serially diluted in sterile PBS and plated on TSA for bacterial enumeration following overnight incubation.

### Biofilm Microtiter Assays

#### Effects on Biofilm Formation

Colonies of either RP73 or PAO1 were inoculated in TSB and cultured for 18–20 h at 37 °C (130 rpm). After incubation, bacterial cultures were centrifuged at 4500 g at 4 °C for 10 min and the pellet washed in PBS. To investigate the effect of MET-1 on growing biofilms, 1 × 10^7^ cfu/mL were treated with either vehicle control (PBS), MET-1 (2, 5 and 10 mg/L), or MET-1 in the presence of tobramycin (4x MIC) and allowed to develop as biofilms in a sterile flat well polycarbonate 96-well plate (200µL/well). The highest MET-1 concentration corresponded to final DMSO concentration of 0.10% (v/v), with matched DMSO-only controls included. Plates were sealed with a breathable membrane and incubated in a humidified chamber at 37 °C for 6, 24 and 48 h under static conditions.

#### Effects on Established Biofilms

200µL of 1 × 10^7^ cfu/mL were added to sterile flat well polycarbonate 96-well plates, sealed with a breathable membrane and incubated in a humidified chamber at 37 °C. To enable detailed analysis of biofilm reduction in metabolic activity/biomass a concentration-response curve was created for tobramycin mediated biofilm response using 0, 0.25, 0.5, 1, 5, 20, 50, 100, 200, 400 and 800x MIC tobramycin. To investigate the potentiating capabilities of MET-1, at 6, 24 and 48 h of biofilm growth, media was carefully removed and the biofilms washed with sterile PBS to remove any remaining planktonic bacteria. The media was replaced with TSB supplemented with either vehicle (PBS), tobramycin (0-800x MIC), MET-1 (2, 5, 10 mg/L) or combinations thereof. Plates were sealed with a breathable membrane and incubated in a humidified chamber at 37 °C for a further 24 h. After 24 h, biofilm-associated effects were quantified as either changes in biomass, metabolic activity or absolute colony-forming units (cfu) quantification within biofilms.

#### Biofilm Biomass Quantification

Biofilm biomass was determined through absorbance at 570 nm of crystal violet (CV) staining as previously described [25]. At each biofilm incubation timepoint, media was removed, and biofilms were carefully washed three times with PBS to remove any non-biofilm resident bacteria. Biofilms were fixed by heat at 60 °C for 1 h on a hot plate and stained for 15 min with 150µL of 2.3% CV in 20% ethanol at room temperature. After staining, excess CV was removed and the biofilm washed three times with PBS. Wells were air-dried, and biofilm bound CV resolubilized with 200µL of 33% (v/v) glacial acetic acid for 1 h at room temperature. Contents of wells were transferred to a new plate and CV absorbance was measured at 570 nm using a microplate spectrophotometer with 33% acetic acid in water as a blank. Preliminary experiments confirmed that MET-1 alone at 2–10 mg/L did not interfere with either resazurin/resorufin fluorescence or crystal violet staining. This was consistent with previous studies using comparable MET-1 concentrations without evidence of assay interference[25].

#### Bacterial Metabolic Activity Quantification

Metabolic activity of all biofilm-associated bacteria was determined using resazurin. At each biofilm incubation timepoint, media was removed and the biofilms were washed three times with sterile PBS to remove any non-biofilm resident bacteria. Biofilms and sterility controls (TSB only) were incubated with 10 µg/mL resazurin in TSB until the control wells sufficiently turned pink. Incubation time varied between 1 and 6 h depending on strain and biofilm age; however, all treatment groups, controls and replicates within an individual plate were incubated with resazurin for the same duration. Resorufin fluorescence was measured at a wavelength of 590 nm with excitation wavelength of 550 nm.

#### Bacterial Viability of Biofilm-Associated Bacteria

Bacterial burden within biofilms was assessed by quantifying cfu using agar plating. At each time point, media was removed and the biofilms washed three times with PBS to remove any non-biofilm resident bacteria. 200µL of sterile PBS was added to each well and the plate was sealed. Biofilms were removed from the wells using sonication for 3 min at high intensity as previously reported [31]. Preliminary experiments confirmed 3 min of sonication did not significantly alter bacterial numbers (Supplementary Fig. 2). Aliquots were serially diluted with sterile PBS and plated onto TSA plates. Plates were subsequently incubated at 37 °C for 16–18 h, and cfu counted.

### Murine Model of Pulmonary Infection

Monotherapies of tobramycin, erdosteine or combinations thereof were assessed using a murine model of chronic pulmonary infection with *P. aeruginosa* as previously described [31, 32]. All procedures were performed in accordance with protocols approved by the Ospedale San Raffaele Institutional Animal Care and Use Committee (IACUC; protocol no: 1438) and authorised by the Italian Ministry of Health guidelines in compliance with national guidelines for the care and use of experimental animals. Immunocompetent male C57BL/6 NCrlBR mice (8–10 weeks, Charles River Laboratories, Calco, Italy) were challenged by intratracheal administration with 1 × 10^6^ cfu/mouse of *P. aeruginosa* RP73 embedded in agar beads. Agar-bead preparation, anaesthesia, and intratracheal infection were performed as previously described [32]. Mice were randomly allocated to experimental groups and balanced according to body weight to obtain similar mean body weights across groups. The order in which groups underwent infection and treatment was varied to minimise potential bias due to operator fatigue. Infections, treatments, and euthanasia were performed in the late morning. Mice received vehicle controls, tobramycin (40 or 160 mg/kg tobramycin via subcutaneous administration), erdosteine (100 mg/kg *p.o*) or tobramycin plus erdosteine (40 and 100 mg/kg respectively). Tobramycin was formulated in water and administered in a volume of 100 µl, whereas erdosteine was formulated in DMEM buffered to pH 7.4 and administered by oral gavage at 10 ml/kg according to individual body weight. The first dose of both erdosteine and tobramycin was administered 5 min before infection, followed by once-daily treatment for 10 consecutive days, including the day of infection. Mice were monitored daily for body weight and clinical signs; no mice reached the predefined humane endpoints (> 20% bodyweight loss and/or severe clinical signs). Three mice died in associated with the experimental procedure; no treatment-related toxicity was observed. At the experimental endpoint, 24 h after the last treatment, euthanasia, bronchoalveolar lavage (BAL), lung collection, and bacterial burden quantification was performed as previously described [32]. CFU enumeration was performed blinding to treatment group. The limit of detection was 200 cfu/lung, corresponding to one colony recovered from the undiluted sample; samples with no detectable colonies were assigned a value of 0 cfu/lung. A cut-off of > 1,000 cfu/lung was used to define chronic infection, as previously described [33]. No additional exclusion criteria were applied. Data were obtained from 1 to 2 independent experiments, depending on the treatment group.

### Data Presentation and Analysis

Data and statistical analysis were performed with GraphPad Prism (GraphPad Software, Inc, San Diego, CA, USA). MIC values are presented as modal values of three repeats performed in triplicate. Time-kill analyses of data between 0 and 24 h are presented as log_10_ mean cfu/mL ± standard error of the mean (SEM). A ≥ 2-log₁₀ cfu/mL reduction between the most active monotherapy and combination therapy was determined as synergistic as previously reported [34]. To complement this threshold-based assessment, drug interactions were also analysed using the Bliss-Loewe Uncertainty Range (BLUR) model as previously described [34]. BLUR defines an expected additive range bounded by Bliss independence and Loewe additivity. Bliss additivity is defined as *E*_AB_= *E*_A_ + *E*_B_ - *E*_A_*E*_B_, where *E*_A_ and *E*_B_ are the fractional effects of tobramycin and MET-1 monotherapy, respectively. Loewe additivity is based on *C*_A_/*C*_A, x_ + *C*_B_/*C*_B, x_ = 1, where *C*_A, x_ and *C*_B, x_ are the concentrations producing an equivalent effect *x*. Combination effects outside the BLUR range were classified as synergistic or antagonistic (*P* < 0.05), while effects within the range were considered additive [34]. Raw cfu/mL data from 3 to 5 independent cultures were analysed using a pre-configured Excel model. BLUR analysis was performed at the 24-hour endpoint for each tobramycin concentration independently, and the resulting observed effect, BLUR lower and upper additive limits, interaction classification and statistical significance were reported for each condition. The limit of detection (LOD) of the time-kill assay was found to be 2 log_10_ cfu/mL (100 cfu/mL) as previously reported [30].

Concentration-response curves were fitted in GraphPad Prism using a four-parameter logistic nonlinear regression model with log₁₀-transformed antibiotic concentration as the independent variable (“Sigmoidal, 4PL, X is log(concentration)”). Curves were fitted to pooled replicate data, with each replicate Y value treated as an individual data point and no outlier exclusion or weighting applied. The lower asymptote was constrained to > 0, while the upper asymptote, Hill slope and log₁₀IC₅₀ were unconstrained. Prism-derived IC₅₀ values are reported in mg/L, while log₁₀ (IC₅₀/MIC) values were used for statistical comparison where indicated. Profile-likelihood 95% confidence intervals were calculated for fitted IC₅₀ values. Biofilm biomass and metabolic activity are expressed as a percentage of the control to account for inherent variability in biofilm growth between cultures [25]. Significant differences in biofilm biomass or metabolic activity after 6, 24 and 48 h were determined using an ordinary two-way ANOVA followed by Tukey’s test (ns, *P* > 0.05; * *P* ≤ 0.05; ** *P* ≤ 0.01; *** *P* ≤ 0.001; and **** *P* ≤ 0.0001). The experimental unit for statistical analysis was the independent bacterial culture. Technical replicate measurements were averaged to generate a single value for each independent culture and these were used for statistical analysis. The minimum reliable signal (MRS), which is the smallest signal level that can be distinguished from background noise with acceptable accuracy and confidence.

$$\boldsymbol{B}^{-}+3\boldsymbol{\sigma}$$

$\boldsymbol{B}^{-}$ = mean blank absorbance, $\boldsymbol{\sigma}$ = standard deviation of blanks.

The same calculation was applied separately to crystal violet absorbance and resazurin fluorescence data. For the crystal violet assay, MRS was expressed as absorbance at 570 nm (OD₅₇₀), whereas for the resazurin assay it was expressed as relative fluorescence units (RFU). This gave MRS values of 0.17 OD₅₇₀ and 5719 RFU, respectively.

For in vivo studies, lung cfu data are presented as median with interquartile range (IQR). Owing to the small and unequal group sizes, normality testing was not considered informative and pulmonary bacterial burden was analysed using a non-parametric Kruskal-Wallis test followed by Dunn’s multiple-comparisons test for selected pairwise comparisons. Bacterial clearance was analysed as a categorical endpoint, with animals classified as cleared (< 1 × 10³ cfu/lung) or chronically infected (≥ 1 × 10³ cfu/lung). Clearance is reported as the number of animals meeting the clearance criterion over the total number of animals in each group, together with exact 95% binomial confidence intervals. Pairwise differences in clearance proportions were assessed using two-sided Fisher’s exact tests.

## Results

### MET-1 Does Not Affect Growth/No-Growth Threshold in the MIC Assay

MIC values for tobramycin alone and in combination for RP73 and PAO1 are shown in Table 1. Based on a clinical breakpoint for tobramycin of 2 mg/L [35], RP73 is classified as tobramycin-resistant (4 mg/L) and PAO1 tobramycin-sensitive (1 mg/L), according to EUCAST Clinical Breakpoint Tables, version 16.1 (2026). Combination with MET-1 did not show any change in tobramycin MIC for either of the *P. aeruginosa* strains, RP73 or PAO1. In line with previous work, MET-1 monotherapy did not achieve a growth/no-growth threshold in the MIC assay at the highest soluble concentration of MET-1 (1024 mg/L). As MET-1 does not achieve a growth/no growth interface, it was not possible to calculate any FICI values for tobramycin in the presence of MET-1 and therefore no additive/synergistic interactions were detected using the chequerboard assay of synergy. For both RP73 and PAO1, combination with erdosteine was determined as being indifferent based on a FICI of 1.5.

**Table 1:**
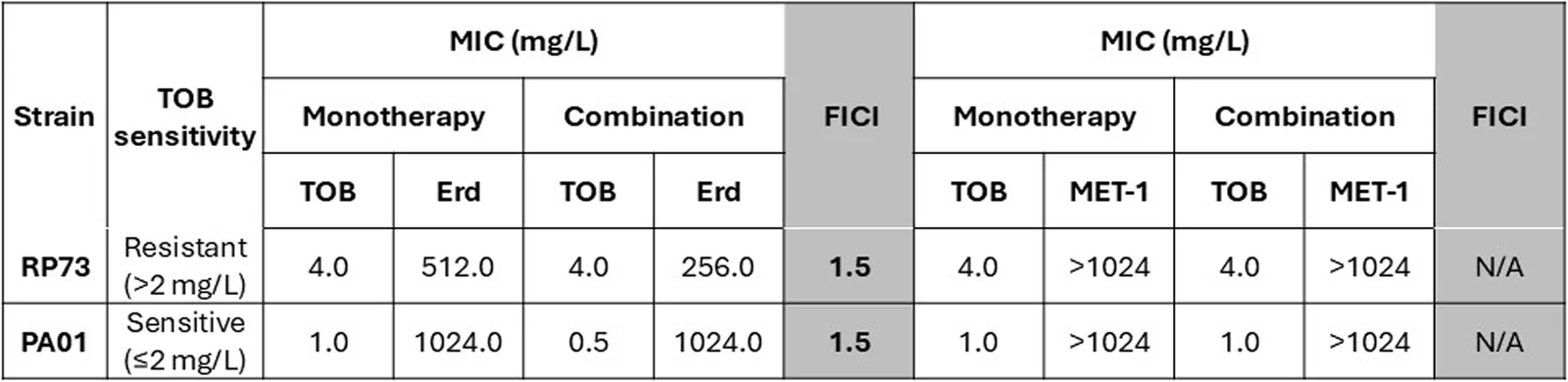
MIC (mg/L) values of tobramycin (TOB), MET-1 and erdosteine (Erd) alone, and the combination of TOB with MET-1 and Erd for RP73 and PAO1. MICs presented as the modal value of three repeats performed in triplicate. Fractional Inhibitory Concentration indices (FICI) were calculated using the concentrations at the growth/no growth interface

### MET-1 Potentiates Tobramycin Against Planktonic RP73 and PAO1 in Vitro

**Fig. 1:**
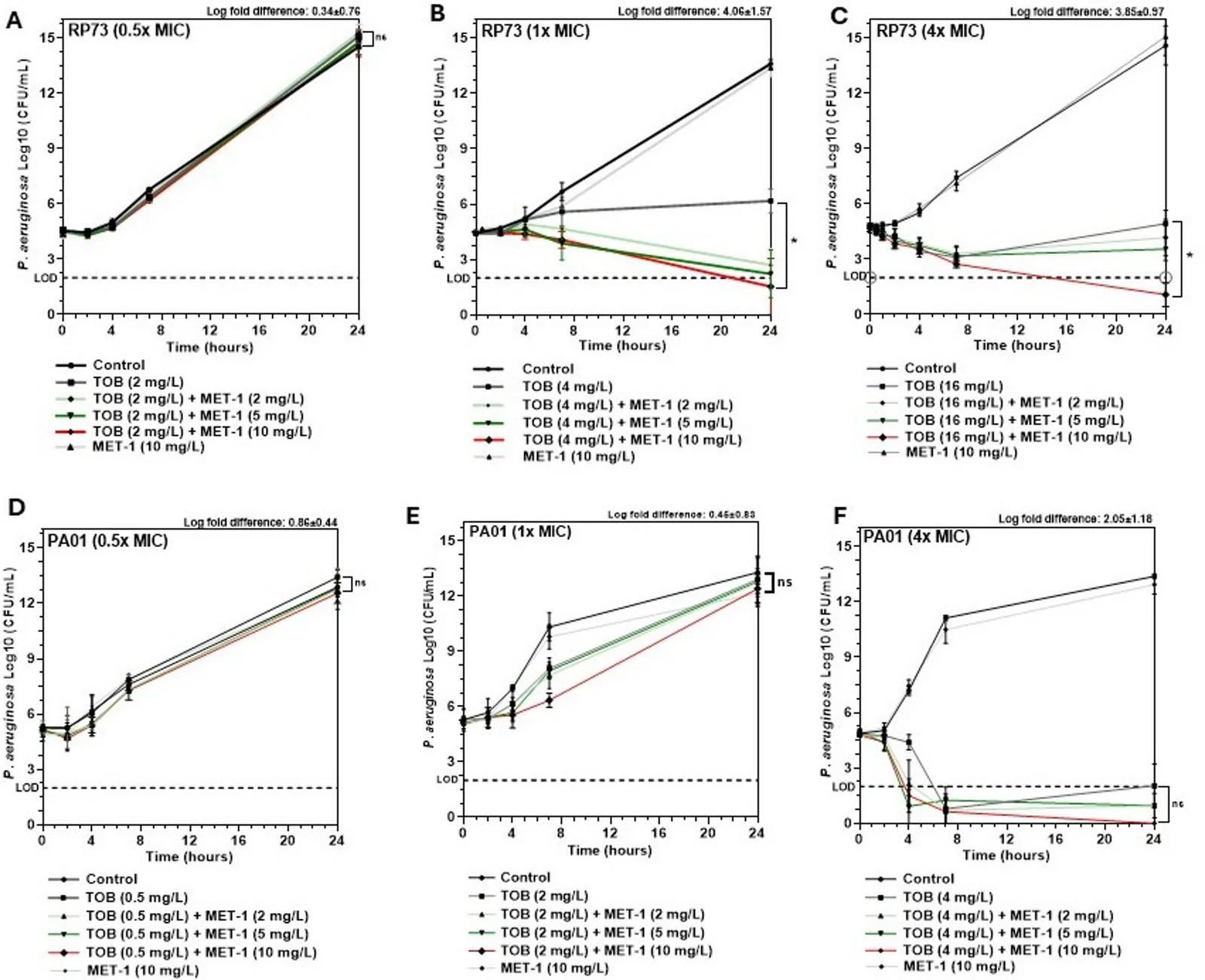
Bacterial time-kill analyses of RP73 and PAO1 using tobramycin at concentrations 0.5x, 1x, and 4x the MIC. Tobramycin MICs were 4 mg/L and 1 mg/L for RP73 (**A-C**) and PAO1 (**D-F**), respectively. Aliquots from the control (black), tobramycin monotherapy (dark grey), MET-1 (10 mg/L) monotherapy (light grey) and tobramycin in combination with 2 mg/L (light green), 5 mg/L (dark green) and 10 mg/L (red) MET-1 were collected for enumeration at 0-, 2-, 4-, 7- and 24-hours post-treatment. Statistical significance of the drug interaction of the combination (tobramycin + MET-1 10 mg/L) compared to tobramycin monotherapy at 24 h was determined using the BLUR model of synergy and indicated with a * (*P* < 0.05) or ns (not significant). Limit of detection (LOD) is marked with a dotted line at 2Log_10_ cfu/mL(100 cfu/mL). Data presented as mean ± SEM log cfu/mL, *N* = 3–5 per group. Each n represents an independent bacterial culture

Combinations of tobramycin and MET-1 were assessed in a bacterial time-kill assay, as this approach captures dynamic changes in bacterial viability over time. We analysed the ability of 10 mg/L MET-1 to potentiate the antimicrobial activity of tobramycin at 0.5x, 1x, and 4x MIC for both RP73 (Fig. 1A-C) and PAO1 (Fig. 1D-F). The concentration of MET-1 used (10 mg/L) is within the same order of magnitude as the peak plasma levels achievable after standard therapeutic dosing with erdosteine. MET-1 exhibited no bactericidal activity against planktonic cultures of RP73 or PAO1, as evidenced by the absence of any significant differences between the untreated control and MET-1 monotherapy at 24 h. Furthermore, no potentiation of tobramycin was observed with MET-1 at sub-therapeutic antibiotic concentrations (0.5x MIC, Fig. 1A). However, MET-1 (10 mg/L) demonstrated synergy with tobramycin at 1x and 4x MIC against RP73 at 24 h, shown by a log_10_-order reduction in bacterial cells of 4.06 ± 1.57 cfu/ml (Fig. 1B, *P* < 0.05) and 3.85 ± 0.97 cfu/mL (Fig. 1C, *P* < 0.05), respectively, compared to tobramycin alone exceeding the threshold for synergy of a ≥ 2-log fold reduction between combination treatment and most active monotherapy. Synergy for both tobramycin concentrations was confirmed by secondary BLUR analysis (*P* < 0.05). For PAO1, no potentiation was observed at 0.5x or 1x MIC (Fig. 1D & E), whilst combination with MET-1 (10 mg/L) showed synergy with tobramycin at 4x MIC at 4 h with a log_10_-order reduction of 3.72 ± 0.79 cfu/mL (Fig. 1F, *P* < 0.05) compared to tobramycin alone, whilst this effect was lost at 24 h. Secondary BLUR analysis revealed synergistic interactions at 4x MIC (*P* < 0.05).

### MET-1 Delays Biofilm Formation in Vitro

Early biofilm formation represents a clinically important window during which antimicrobial intervention may prevent the transition to persistent, treatment-refractory infections. Given the observed potentiation by MET-1 in planktonic cultures, we next evaluated the impact of MET-1 on biofilm resident cultures. In agreement with previous research indicating that MET-1 can reduce bacterial adhesion, we demonstrated that treatment with MET-1 significantly reduced biofilm biomass for three different *S. aureus* strains (USA300, ATCC 33591 and ATCC 25923) at 6 h supporting the concept that MET-1 may be reducing early biofilm development through reduced bacterial adhesion (Supplementary Figs. 1 A-C). Significantly, we did not see this effect with the gram-negative *P. aeruginosa* strains RP73 or PAO1 (Supplementary Fig. 1D &E).

**Fig. 2:**
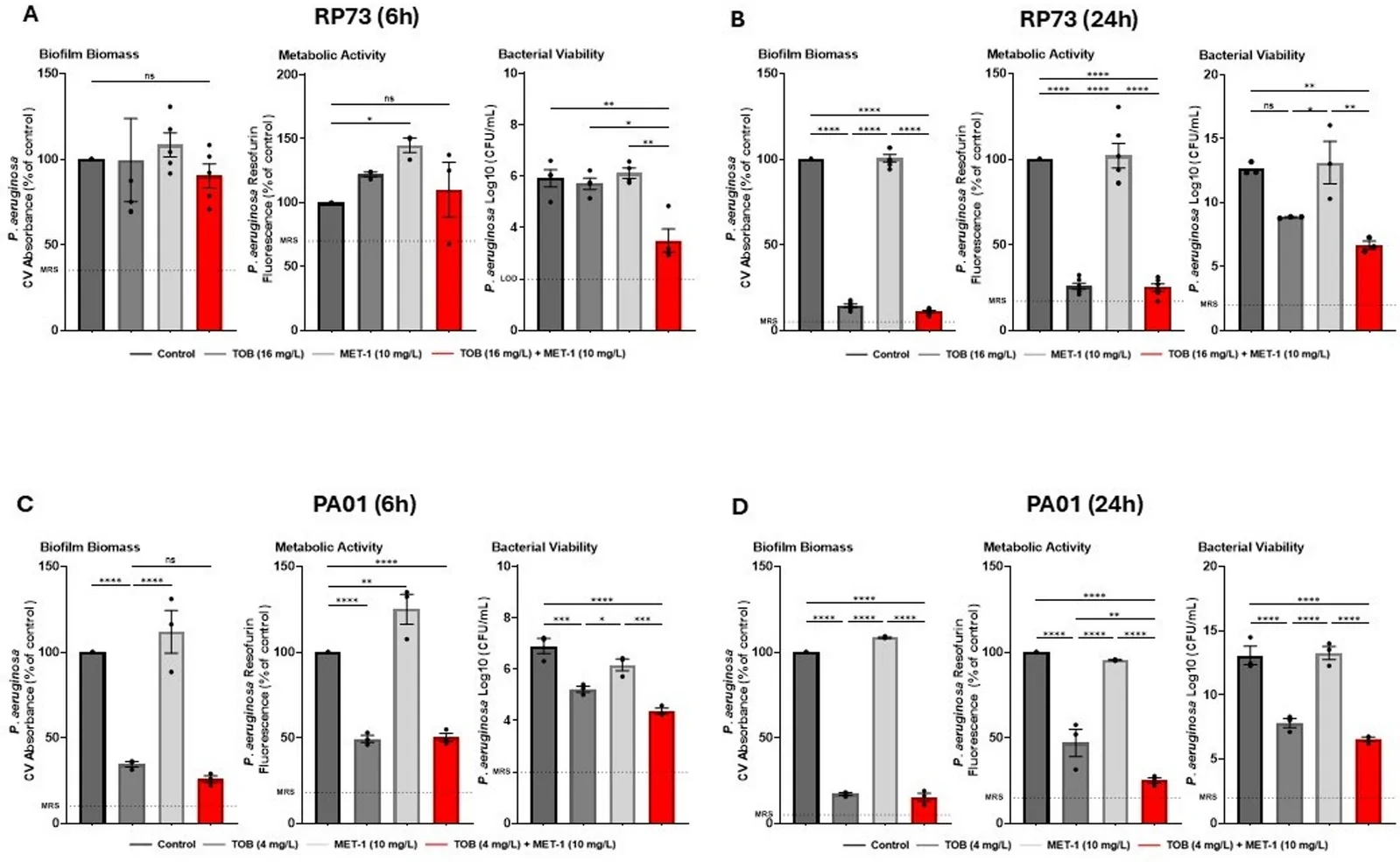
Effect of MET-1 on tobramycin activity in developing biofilms as measured by biofilm biomass, metabolic activity and bacterial viability of biofilm-associated cells. RP73 and PAO1 biofilms were grown in the presence of vehicle (black), tobramycin at 4x MIC (dark grey), MET-1 (10 mg/L) (light grey), or tobramycin at 4x MIC in combination with 10 mg/L (red) MET-1. Tobramycin MICs were 4 mg/L and 1 mg/L for RP73 (**A + B**) and PAO1 (**C + D**), respectively. Biofilms were assessed following 6 and 24 h of incubation at 37 °C under static conditions. Biofilm biomass was measured by crystal violet staining, metabolic activity through the formation of resorufin and bacterial content by quantification. Crystal violet and resorufin data are presented as percentage of control. Minimum reliable signal (MRS) as a percentage of control and limit of detection (LOD) is marked with a dotted line. Treatment groups were compared using an ordinary 1-way ANOVA followed by Tukey’s multiple comparison test (ns, *P* > 0.05; * *P* ≤ 0.05; ** *P* ≤ 0.01; *** *P* ≤ 0.001; and **** *P* ≤ 0.0001). Data presented as mean ± SEM, *N* = 3–5 per group. Each n represents an independent bacterial culture, calculated as the mean of two technical replicates

We next investigated whether combining tobramycin with MET-1 (10 mg/L) could alter biofilm formation. At 6–24 h of development, no significant difference was observed in either biofilm biomass or metabolic activity of RP73 biofilms grown in the presence of 4x MIC tobramycin monotherapy, or those combined with MET-1 at any tested concentration (Fig. 2A & B). In young biofilms (6 h) tobramycin in combination with 10 mg/L MET-1 significantly reduced total recovered bacterial numbers with a log_10_-order reduction in cfu/mL of 1.63 ± 0.24 (*P* < 0.05) (Fig. 2A). However, this reduction was not seen at 24 h (Fig. 2B), potentially indicating delayed biofilm formation through reduced bacterial adhesion. Similarly, for PAO1 biofilms, no significant differences were seen for biofilm biomass for biofilms grown in the presence of tobramycin (4x MIC) compared to those grown in the presence of tobramycin (4x MIC) in combination with MET-1 (10 mg/L) at either 6–24 h. However, PAO1 biofilms treated with 4x MIC tobramycin and MET-1 revealed a significant reduction in metabolic activity at 24 h compared to tobramycin alone (46.6%±8.7% (*P* < 0.01). Whilst metabolic activity was reduced at 24 h, this was not reflected by any changes in bacterial content within the biofilms (Fig. 2D). This suggests that tobramycin: MET-1 combination therapy may delay the formation of biofilms, although there is no evidence for potentiation in tobramycin mediated reduction in *P. aeruginosa* bacterial burden within the biofilm.

### MET-1 Potentiates Tobramycin Against Established Biofilms of PAO1 and RP73

Whilst it was of interest to investigate the potential of MET-1 to synergise with tobramycin in interfering with biofilm formation, greater clinical relevance lies in the ability to disrupt established biofilms, as bacterial cells are more resistant to conventional antibiotics in established mature biofilms. Using readouts of biofilm size (biomass) and viability (metabolic activity), we characterised a concentration response curve for tobramycin covering 0-800x MIC for antimicrobial activity against RP73 and PAO1 biofilms established for 6, 24 and 48 h prior to drug treatment. We then evaluated the ability of 2, 5 and 10 mg/L MET-1 to induce a leftward shift in tobramycin’s concentration-response curve, quantified as a reduction in the IC_50_ (mg/L) for both biofilm biomass and metabolic activity.

**Fig. 3:**
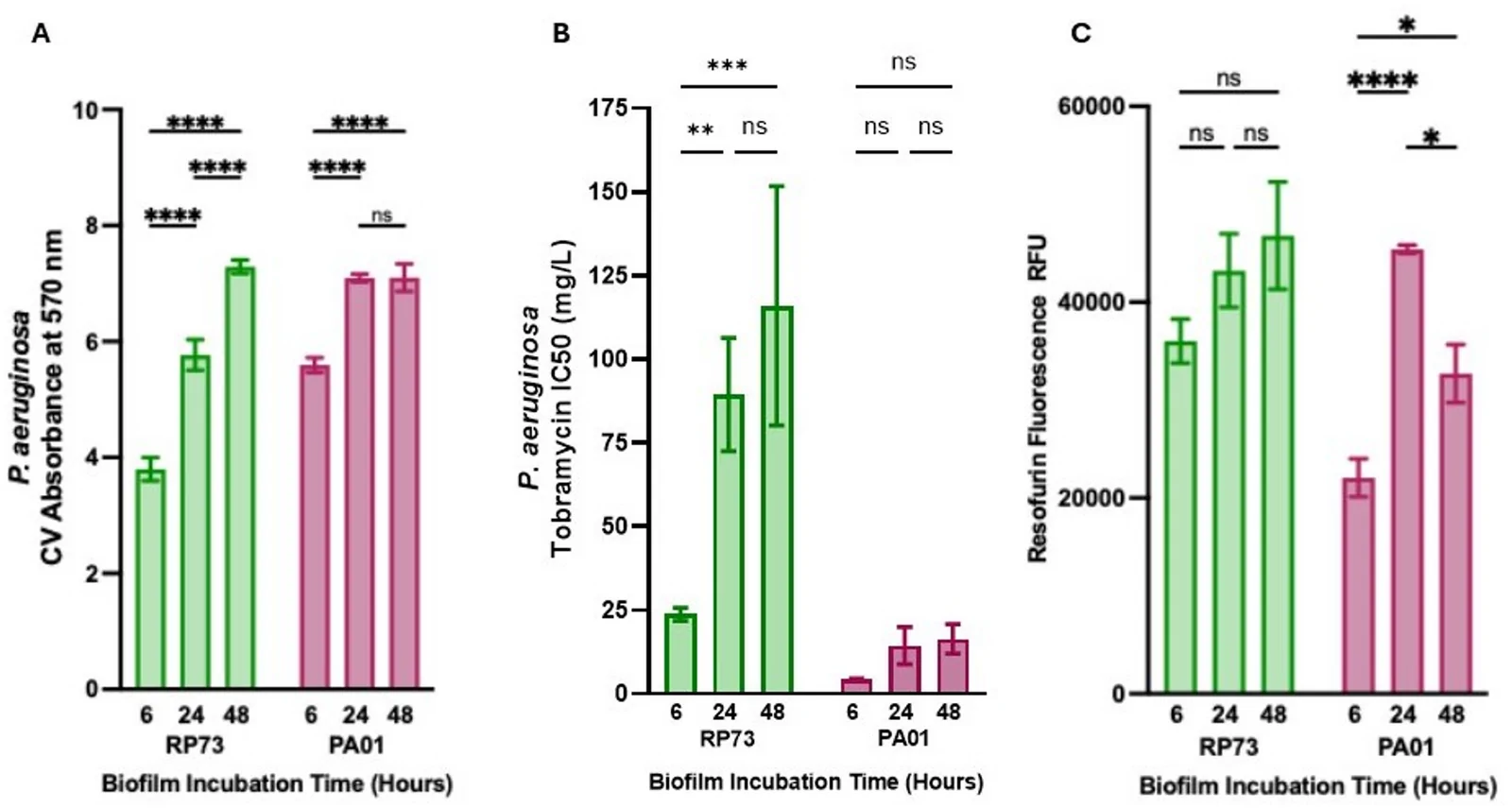
Mature biofilms are associated with increased biomass and reduced sensitivity to tobramycin. Biofilms were left to incubate for up to 48 h and biomass quantified via measurement of crystal violet absorbance at 570 nm. *N* = 3 where each n represents an independent bacterial culture, calculated as the mean of two technical replicates. **A** Biofilm biomass was quantified by crystal violet absorbance at 570 nm. **B** Biofilm metabolic activity was quantified by resazurin reduction and expressed as resorufin fluorescence (RFU). **C** Tobramycin susceptibility was assessed by determining biomass-derived IC_50_ values following treatment of established biofilms. Data are presented as mean ± SEM. Statistical significance was determined by ordinary two-way ANOVA followed by Tukey’s multiple-comparisons test (ns, *P* > 0.05; *P ≤ 0.05; **P ≤ 0.01; ***P ≤ 0.001; ****P ≤ 0.0001). *n* = 3, Each n represents an independent culture, calculated as the mean of two technical replicates

In line with previous research, we confirmed that biofilm maturation was associated with significant increases in biofilm biomass in mature (24, and 48 h) compared to young biofilms (6 h) for both RP73 (6 h: 3.80 ± 0.20; 24 h: 5.77 ± 0.27 *P* < 0.001; 48 h: 7.29 ± 0.12, OD, *P* < 0.001, Fig. 3A) and PAO1 (6 h: 5.59 ± 0.13; 24 h: 7.10 ± 0.07, *P* < 0.001; 48 h: 7.10 ± 0.24 OD, *P* < 0.001, Fig. 3A). This was also reflected with corresponding increases in metabolic activity (Fig. 3B). As increased biofilm maturation is associated with decreased antibiotic efficacy, we next assessed the susceptibility of young and mature biofilms to tobramycin.

Young RP73 biofilms were more susceptible to tobramycin therapy compared to mature biofilms with significant increases in the IC_50_ (mg/L) for tobramycin at 24 and 48 h compared to 6 h for biomass (6 h: 23.75 ± 1.83 mg/L; 24 h: 74.53 ± 20.32 mg/L, *P* < 0.05; 48 h: 86.94 ± 38.45 mg/L, *P* < 0.01, Fig. 3C). Similarly, tobramycin IC_50_ values were significantly elevated in mature 48-hour PAO1 biofilms compared to young 6-hour biofilms for biomass (6 h: 4.32 ± 0.24 mg/L; 24 h: 14.42 ± 5.57 mg/L; 48 h: 16.38 ± 4.41 mg/L, *P* < 0.05, Fig. 3C). Although the underlying mechanism was not directly investigated here, these findings are consistent with previous literature which demonstrated reduced antimicrobial penetration in established biofilms due to increased production of extracellular polymeric substance matrix.

**Fig. 4:**
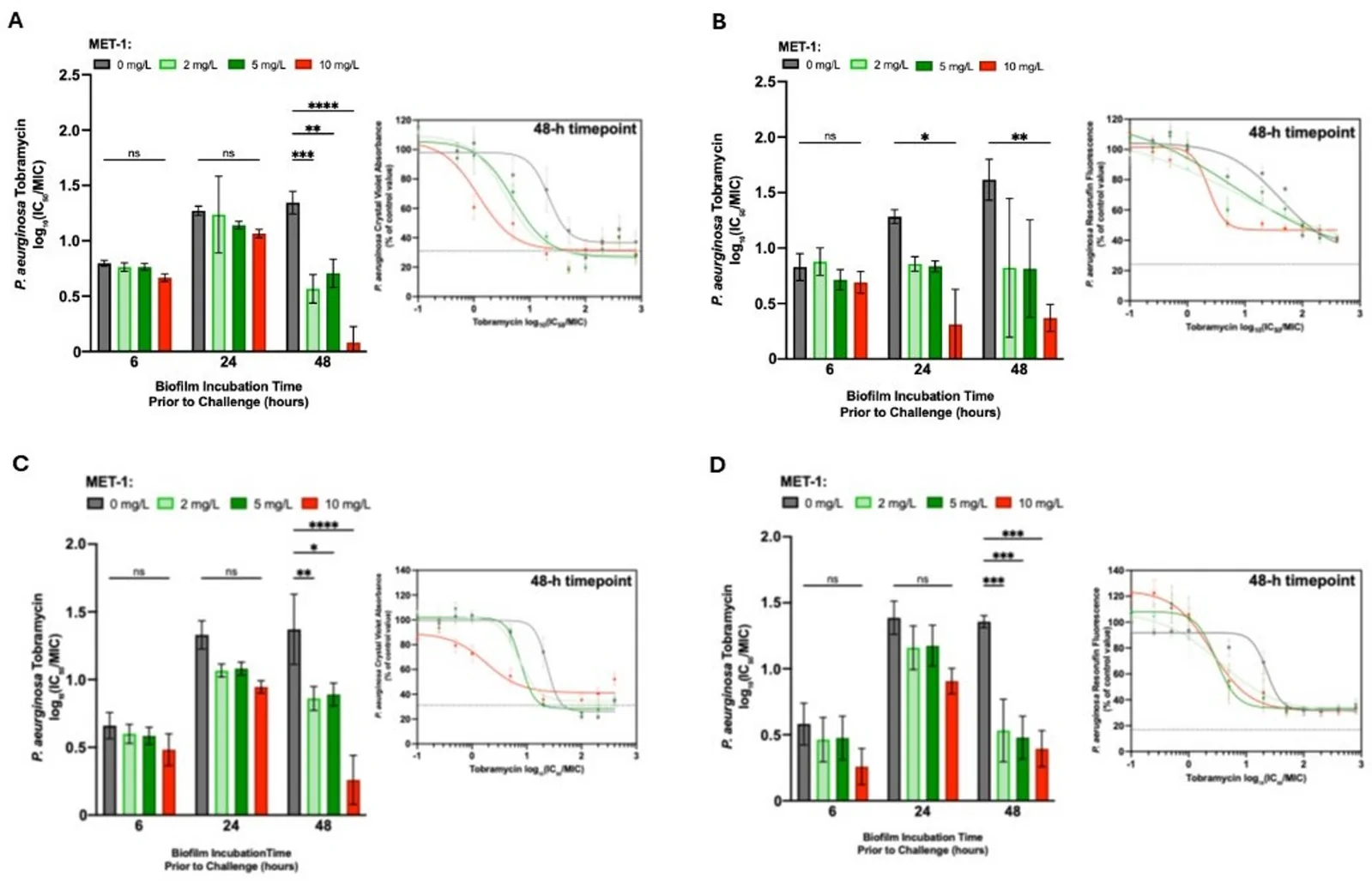
Effect of MET-1 on tobramycin activity in established biofilms as measured by biofilm biomass and metabolic activity. Schematics illustrating changes in biofilm biomass (**A **&** C**) and metabolic activity (**B **&** D**) of established biofilms of RP73 (**A **&** B**) and PAO1 (**C **&** D**) treated with tobramycin 0-800x MIC either alone (grey) or in combination with 2 mg/L (light green), 5 mg/L (dark green) or 10 mg/L (red) MET-1. Tobramycin MICs were 4 mg/L and 1 mg/L for RP73 and PAO1, respectively. Following treatment at either 6, 24–48 h, biofilms were left to incubate for a further 24 h. Tobramycin concentrations were normalised to the strain-specific MIC prior to log₁₀ transformation and nonlinear regression. Significant differences in biofilm biomass or metabolic activity after 6, 24 and 48 h were determined using an ordinary two-way ANOVA followed by Tukey’s test (ns, *P* > 0.05; * *P* ≤ 0.05; ** *P* ≤ 0.01; *** *P* ≤ 0.001; and **** *P* ≤ 0.0001). Minimum reliable signal (MRS) is marked with a dotted line. Each n represents an independent bacterial culture, calculated as the mean of two technical replicates

Combination of tobramycin with 10 mg/L MET-1 in mature biofilms (48 h) induced a statistically significant decrease in the log₁₀(IC₅₀/MIC) for tobramycin, which was seen in both biofilm biomass (RP73: Veh 1.34 ± 0.10 vs. 10 mg/L MET-1: 0.08 ± 0.14 log₁₀(IC₅₀/MIC), *P* < 0.0001; PAO1: Veh 1.37 ± 0.26 vs. 10 mg/L MET-1: 0.26 ± 0.18 log₁₀(IC₅₀/MIC), *P* < 0.001) and metabolic activity (RP73: Veh 1.62 ± 0.18 vs. 10 mg/L MET-1: 0.37 ± 0.12 log₁₀(IC₅₀/MIC), *P* < 0.01; PAO1: Veh 1.36 ± 0.05 vs. 10 mg/L MET-1: 0.39 ± 0.14 log₁₀(IC₅₀/MIC), *P* < 0.001, Fig. 4). For both RP73 and PAO1, this was not seen at 6 h.

### MET-1 Enhances Tobramycin in a Murine Model of Chronic RP73 Infection

**Fig. 5:**
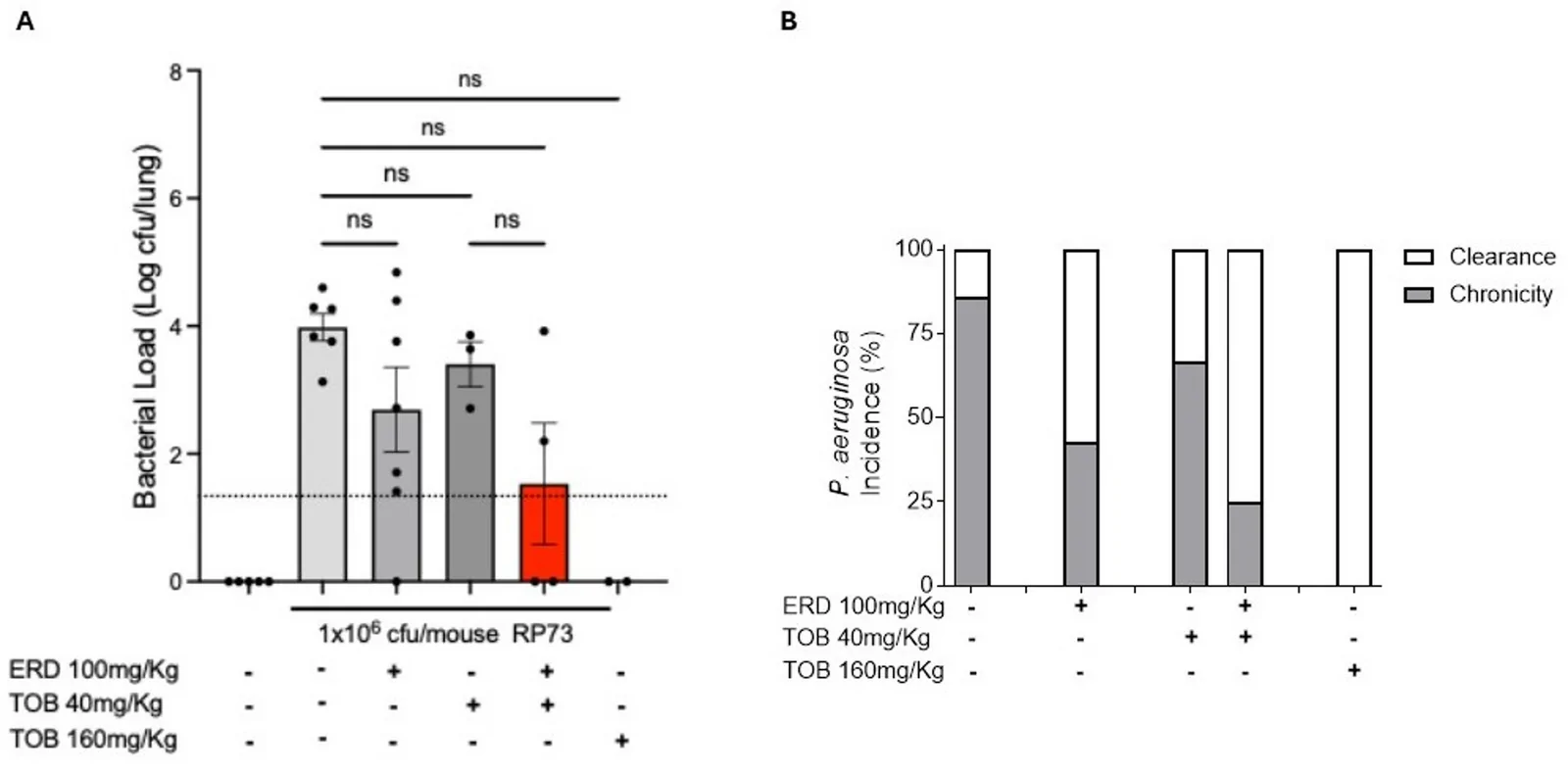
Effects of combination therapy of tobramycin and erdosteine in a murine model of chronic pulmonary infection with RP73. C57Bl/6 mice (aged 8–10 weeks) received intratracheal inoculation with 1 × 10^6^ cfu/mouse RP73 embedded in agar beads. Animals received daily therapy with either vehicle or 40 mg/kg tobramycin *s.c.* in the presence or absence of 100 mg/kg erdosteine *p.o.* 160 mg/kg tobramycin monotherapy was also used as a positive control demonstrating complete eradication. Animals were monitored daily throughout the duration of the study. 10 days post infection, mice were killed, the lungs excised, homogenised and plated on tryptic soy agar to determine the bacterial burden (**A**), whilst data were also expressed as the percentage of animals clearing the pulmonary infection between each treatment group. The limit of detection of bacterial enumeration was 220 cfu/lung (dotted line) corresponding to one colony recovered from the undiluted samples, samples with no detectable colonies were assigned a value of 0 cfu/lung. Data were analysed using a non-parametric Kruskal–Wallis test followed by Dunn’s multiple-comparisons test. **B** A cut off of 1000 cfu/lung was used to distinguish bacterial clearance from chronic colonisation. Group sizes were vehicle, *n* = 7; erdosteine 100 mg/kg, *n* = 7; tobramycin 40 mg/kg, *n* = 3; erdosteine 100 mg/kg plus tobramycin 40 mg/kg, *n* = 4; and tobramycin 160 mg/kg, *n* = 2. Bacterial clearance was analysed as a categorical endpoint, with animals classified as cleared (< 1 × 10³ cfu/lung) or chronically infected (≥ 1 × 10³ cfu/lung). Pairwise differences in clearance proportions were assessed using two-sided Fisher’s exact tests

Having identified that combining MET-1 with tobramycin enhances antimicrobial activity in both planktonic cultures and established biofilms, we advanced our drug combination to an established murine model of chronic infection with *P. aeruginosa* strain RP73 [31, 32, 36]. Mice were infected with 1 × 10^6^ cfu/mouse *P. aeruginosa* embedded in agar beads and monitored for 10 days. After infection, animals were treated daily with either vehicle control, 100 mg/kg erdosteine via oral gavage (*p.o.*), 40 or 160 mg/kg tobramycin subcutaneously (*s.c.)* or combinations thereof. Infection with RP73 resulted in persistent pulmonary bacterial burden at Day 10, with vehicle-treated animals exhibiting a median bacterial load of 4.06 log₁₀ cfu/lung [IQR 3.78–4.29] (Fig. 5A). Treatment with 160 mg/kg tobramycin resulted in complete bacterial clearance by Day 10. Consistent with the in vitro findings, erdosteine showed limited activity as a monotherapy, with bacterial burden remaining variable following treatment with 100 mg/kg erdosteine and no significant difference compared with vehicle-treated controls. Similarly, treatment with 40 mg/kg tobramycin produced only a modest reduction in bacterial burden relative to vehicle, which was not statistically significant (Fig. 5A).

The greatest reduction in pulmonary bacterial burden was observed following combination treatment with 100 mg/kg erdosteine and 40 mg/kg tobramycin, for which the median bacterial load was 1.10 log₁₀ cfu/lung [IQR 0–2.63]. However, owing to the small and unequal group sizes, this did not identify a statistically significant difference between combination treatment and vehicle or between combination treatment and 40 mg/kg tobramycin monotherapy. Nevertheless, the lower bacterial burden observed in the combination group indicated a greater numerical reduction than with either component alone (Fig. 5A).

Supporting the greater reduction in pulmonary bacterial burden observed with combination treatment, bacterial clearance was additionally analysed as a categorical endpoint (Fig. 5B) Clearance, defined as a pulmonary bacterial burden < 1 × 10³ cfu/lung, was observed in 1/7 vehicle-treated animals (14.3%), 4/7 animals receiving 100 mg/kg erdosteine (57.1%), 1/3 receiving 40 mg/kg tobramycin (33.3%), and 3/4 receiving combination treatment (75.0%). Both animals receiving 160 mg/kg tobramycin met the clearance criterion (2/2; 100%). Although the proportion of animals achieving clearance was numerically greater following combination treatment than following vehicle or low-dose tobramycin monotherapy, these differences did not reach statistical significance by Fisher’s exact test.

In summary, all these data support our in vitro findings demonstrating an ability of erdosteine to may enhance the antimicrobial activity of tobramycin in a murine model of chronic infection with *P. aeruginosa.*

## Discussion

Lower respiratory tract infections with gram-negative pathogens such as *P. aeruginosa* are one of the leading causes of death globally and have a particular impact on patients with chronic respiratory diseases such as CF and COPD, where accumulation of hyper concentrated mucus in the airways facilitates biofilm formation and reduces antibiotic activity [37]. Therefore, drugs that improve antibiotic penetration or potentiate antimicrobial activity may offer a valuable strategy to enhance treatment outcomes. The orally active prodrug erdosteine, originally developed as a mucolytic for the treatment of acute and chronic respiratory diseases, has demonstrated pre-clinical and clinical evidence of a multimodal mechanism of action, including mucoactive, antioxidant, anti-inflammatory and antiviral actions [12, 13]. Furthermore, unlike other thiol-based mucolytics such as carbocysteine and NAC which have been shown to inactivate antibiotics, due to the unique chemical structure of erdosteine, research has demonstrated antibiotic-enhancing activities against Gram-positive pathogens [12, 13, 25, 26]. Here we provide evidence that erdosteine also enhances the aminoglycoside antibiotic tobramycin against both drug-sensitive and drug-resistant strains of *P. aeruginosa*.

Whilst *P. aeruginosa* biofilms present a unique clinical challenge, as they are derived from planktonic precursor populations, assessing antimicrobial combinations against both planktonic and biofilm-associated cells is essential to determine their efficacy across distinct physiological states relevant to infection. Bacterial time-kill analyses demonstrated synergistic activity between the primary active metabolite of erdosteine, MET-1 (10 mg/L), and tobramycin against planktonic cultures of the MDR *P. aeruginosa* strain RP73, with combination therapy resulting in significant reductions in viable cell counts when combined with tobramycin at 1x and 4x MIC, respectively (*P* < 0.05). Whilst synergistic activity was also seen in planktonic cultures of the laboratory strain PAO1, synergistic activity of the combination was only observed at 4 h compared to tobramycin alone (*P* < 0.05). Significantly the potentiation of tobramycin against planktonic cultures occurred despite MET-1 exhibiting no intrinsic antibacterial activity, as confirmed by MIC assays showing no reduction in bacterial metabolic activity at the highest soluble concentration tested (1024mg/L). No interaction was observed between MET-1 and tobramycin at sub-MIC concentrations, suggesting that the potentiating effects of MET-1 are restricted to bactericidal concentrations of tobramycin. The redox-active nature of MET-1 may suggest that redox-dependent processes may contribute to its interaction with tobramycin, as bacterial redox state and metabolic activity are known to influence antibiotic efficacy and cellular susceptibility [38].

A significant issue associated with the treatment of chronic *P. aeruginosa* is the propensity for *P. aeruginosa* to form biofilms. As biofilms mature, they transition from a temporary layer of surface-attached bacteria into a highly organised, complex and resilient three-dimensional structures which can impede antibiotic penetration, leading to increased resistance to antimicrobial therapy and necessitating higher doses that may cause renal and hepatic toxicity [39–41]. Combination with 10 mg/L MET-1 demonstrated a significant reduction in the log₁₀(IC₅₀/MIC) of tobramycin in mature biofilms for both strains of *P. aeruginosa*, although this was not seen in young (6 h) or developing biofilms. A key difference between young biofilms is the lack of a defined three-dimensional architecture and reduced complex matrix structures [42], whilst older biofilms often exhibit increased cell density, nutrient and oxygen gradients, and enhanced defence mechanisms such as efflux pump systems and reduced metabolic activity [6, 43]. Combined with an increase in negatively charged biofilm matrix components in mature biofilms, these changes promote electrostatic sequestration of cationic antibiotics, such as tobramycin, reducing their penetration across the biofilm and therapeutic efficacy [44]. This finding was replicated in our own study, where we demonstrated a significant increase in the IC50 of tobramycin in mature biofilms of RP73 and PAO1 compared to young biofilms. The greater potentiating activity of MET-1 in mature compared with younger or developing biofilms is consistent with the hypothesis that MET-1 may modify the extracellular matrix in a manner that facilitates antibiotic activity. MET-1 contains a free thiol group capable of disrupting disulphide bonds in mucus glycoproteins [5], raising the possibility that similar interactions could alter disulphide-dependent components of the biofilm matrix. Consistent with this, addition of 10 mg/L MET-1 reduced tobramycin IC₅₀ values in mature RP73 and PAO1 biofilms, with the greatest effects observed at 48 h (RP73: 6 h vehicle, 25.16 mg/L; 48 h + 10 mg/L MET-1, 4.80 mg/L; PAO1: 6 h vehicle, 4.57 mg/L; 48 h + 10 mg/L MET-1, 1.82 mg/L). These maturity-dependent reductions were statistically significant at selected MET-1 concentrations, supporting a greater effect of MET-1 in established biofilms. However, antibiotic penetration was not directly measured, and these findings therefore do not demonstrate enhanced diffusion through the biofilm matrix. Future studies using direct antibiotic-penetration assays would be required to test this mechanism.

Extending from the in vitro analysis of MET-1 combination therapy with tobramycin we utilised a chronic mouse model of pulmonary infection with *P. aeruginosa*. For the treatment of respiratory diseases such as CB and COPD, the human dose regime is 300 mg twice daily [19]. We therefore calculated the mouse equivalent dose of 100 mg once daily and treated animals for 10 days via oral gavage in line with the clinically approved prescription of erdosteine. Animals were also dosed with tobramycin that was either sub-threshold, or associated with complete clearance (40 or 160 mg/kg) as previously reported [32]. In our studies, 160 mg/kg completely cleared the infection, whilst monotherapy with either 100 mg/kg erdosteine or 40 mg/kg tobramycin failed to demonstrate any significant reduction in pulmonary bacterial load. However, combination of 100 mg/kg erdosteine with 40 mg/kg was associated with a lower pulmonary bacterial burden than vehicle treatment. These results are consistent with previous clinical trials that demonstrated adjunct erdosteine therapy increased sputum β-lactam concentrations, resulting in faster symptom resolution in patients with CB [26]. Furthermore, a systematic review and meta-analysis of seven RCTs in patients with stable or exacerbated CB/COPD confirmed that erdosteine plus antibiotics is more effective than antibiotics alone in treating acute infective exacerbations [27].

In conclusion, MET-1 enhanced selected tobramycin responses against *P. aeruginosa* RP73 and PAO1 under defined in vitro conditions, while erdosteine-containing therapy was associated with a lower pulmonary bacterial burden in the murine RP73 infection model, highlighting the potential therapeutic benefit of adjunct erdosteine therapy in chronic *P. aeruginosa* infections or during acute infective PEx. These findings support further investigation of erdosteine and MET-1 as potential adjuncts to antimicrobial therapy.

## Supplementary Information

Supplementary Material 1: Supplementary Figure 1: Effect of MET-1 alone versus vehicle control on bacterial adhesion at 6, 24 and 48 hours post biofilm formation. Changes in crystal violet absorbance (biofilm biomass) following incubation with vehicle (grey) or MET-1 at a final concentration of 2 mg/L (light green), 5 mg/L (dark green) and 10 mg/L (red) for 6, 24 and 48 hours are presented for biofilms of *S. aureus*: USA300 (**A**), ATCC 33591, (**B**) ATCC 25923, (**C**) and *P. aeruginosa*: RP73 (**D**) and PAO1 (**E**). Data presented of control (%). Minimum reliable signal is marked with a dotted line. Treatment groups were compared using 2-way ANOVA followed by Tukey’s multiple comparison test (ns, *P* > 0.05; * *P* ≤ 0.05; ** *P* ≤ 0.01; *** *P* ≤ 0.001; and **** *P* ≤ 0.0001). Each n represents an independent bacterial culture, calculated as the mean of two technical replicates. Supplementary Figure 2. Effect of sonication duration on recovery of viable bacteria from RP73 and PAO1 biofilms. 6 hour established biofilms of (**A**) RP73 and (**B**) PAO1 were subjected to sonication for 0, 1, 5 or 10 min prior to viable bacterial enumeration by cfu. Sterility controls (TSB only) are shown for comparison. Data are presented as mean ± SEM with individual replicates shown. Statistical significance was assessed by one-way ANOVA with multiple-comparisons testing. ns, not significant; ****P < 0.0001. Supplementary Figure 3. Tobramycin IC_50_ values in established RP73 and PAO1 biofilms following treatment with MET-1. Biofilms were established for 6, 24 or 48 hours and subsequently treated with tobramycin in the absence or presence of 2, 5 or 10 mg/L MET-1. Treatment effects were assessed using crystal violet staining for biofilm biomass and resazurin reduction for metabolic activity. Concentration–response curves were fitted using four-parameter logistic nonlinear regression with log_10_-transformed tobramycin concentration as the independent variable. IC_50_ values are reported in mg/L with 95% profile-likelihood confidence intervals. Individual replicate Y values were included in the pooled nonlinear regression fit. NE, not estimable by profile-likelihood analysis.

## Data Availability

The data presented in this study are available on request from the corresponding authors.
