# Supplementary material for: Erdosteine Enhances Tobramycin Activity Against a Tobramycin-Resistant Clinical Isolate and PAO1 Reference Strain of Pseudomonas aeruginosa
Source: Lung. 2026 Nov 9;204(1):93. doi: 10.1007/s00408-026-00951-6 (PMC13748102; doi:10.1007/s00408-026-00951-6)
Supplement: Supplementary file 1 [file 408_2026_951_MOESM1_ESM.zip › 408_2026_951_MOESM1_ESM.docx]

**Supplementary Figure 1: Effect of MET-1 alone versus vehicle control on bacterial adhesion at 6, 24 and 48 hours post biofilm formation.** Changes in crystal violet absorbance (biofilm biomass) following incubation with vehicle (grey) or MET-1 at a final concentration of 2 mg/L (green), 5 mg/L (blue) and 10 mg/L (purple) for 6, 24 and 48 hours are presented for biofilms of *S. aureus*: USA300 (**A**), ATCC 33591 (**B**), ATCC 25923 (**C**), and *P. aeruginosa*: RP73 (**D**) and PAO1 (**E**). Data presented of control (%). Minimum reliable signal is marked with a dotted line. Treatment groups were compared using 2-way ANOVA followed by Tukey’s multiple comparison test (ns, P > 0.05; * P ≤ 0.05; ** P ≤ 0.01; *** P ≤ 0.001; and **** P ≤ 0.0001). All significant differences between groups are indicated, where there were no significant differences between any group, only the control vs MET-1 at the highest concentration (10 mg/L) is labelled as “ns”. Each n represents an independent bacterial culture, calculated as the mean of two technical replicates.

**Supplementary Figure 2. Effect of sonication duration on recovery of viable bacteria from RP73 and PAO1 biofilms.** 6 hour established biofilms of (A) RP73 and (B) PAO1 were subjected to sonication for 0, 1, 5 or 10 min prior to viable bacterial enumeration by CFU. Sterility controls (TSB only) are shown for comparison. Data are presented as mean ± SEM with individual replicates shown. Statistical significance was assessed by one-way ANOVA with multiple-comparisons testing. ns, not significant; ****P < 0.0001.

**Supplementary Figure 3. Tobramycin IC₅₀ values in established RP73 and PAO1 biofilms following treatment with MET-1.** Biofilms were established for 6, 24 or 48 hours and subsequently treated with tobramycin in the absence or presence of 2, 5 or 10 mg/L MET-1. Treatment effects were assessed using crystal violet staining for biofilm biomass and resazurin reduction for metabolic activity. Concentration–response curves were fitted using four-parameter logistic nonlinear regression with log₁₀-transformed tobramycin concentration as the independent variable. IC₅₀ values are reported in mg/L with 95% profile-likelihood confidence intervals. Individual replicate Y values were included in the pooled nonlinear regression fit. NE, not estimable by profile-likelihood analysis.
